# Rise and fall of Landau’s quasiparticles while approaching the Mott transition

**DOI:** 10.1038/s41467-021-21741-z

**Published:** 2021-03-10

**Authors:** Andrej Pustogow, Yohei Saito, Anja Löhle, Miriam Sanz Alonso, Atsushi Kawamoto, Vladimir Dobrosavljević, Martin Dressel, Simone Fratini

**Affiliations:** 1 1. Physikalisches Institut, Universität Stuttgart, 70569, Stuttgart Germany; 2 Department of Physics and Astronomy, UCLA, Los Angeles, CA USA; 3 Department of Physics, Graduate School of Science, Hokkaido University, Sapporo, Japan; 4 Department of Physics and National High Magnetic Field Laboratory, Florida State University, Tallahassee, FL USA; 5 Institut Néel - CNRS and Université Grenoble Alpes, Grenoble Cedex 9, France; 6 Present Address: Institute of Solid State Physics, Vienna University of Technology, Vienna, Austria

**Keywords:** Electronic properties and materials, Phase transitions and critical phenomena, Quantum fluids and solids, Theoretical physics

## Abstract

Landau suggested that the low-temperature properties of metals can be understood in terms of long-lived quasiparticles with all complex interactions included in Fermi-liquid parameters, such as the effective mass *m*^⋆^. Despite its wide applicability, electronic transport in bad or strange metals and unconventional superconductors is controversially discussed towards a possible collapse of the quasiparticle concept. Here we explore the electrodynamic response of correlated metals at half filling for varying correlation strength upon approaching a Mott insulator. We reveal persistent Fermi-liquid behavior with pronounced quadratic dependences of the optical scattering rate on temperature and frequency, along with a puzzling elastic contribution to relaxation. The strong increase of the resistivity beyond the Ioffe–Regel–Mott limit is accompanied by a ‘displaced Drude peak’ in the optical conductivity. Our results, supported by a theoretical model for the optical response, demonstrate the emergence of a bad metal from resilient quasiparticles that are subject to dynamical localization and dissolve near the Mott transition.

## Abstract (web summary)

Charge transport in strongly correlated electron systems is not fully understood. Here, the authors show that resilient quasiparticles at finite frequency persist into the bad-metal regime near a Mott insulator, where dynamical localization results in a ‘displaced Drude peak’ and strongly enhanced dc resistivity.

## Introduction

Conduction electrons in solids behave differently compared to free charges in vacuum. Since it is not possible to exhaustively model the interactions with all constituents of the crystal (nuclei and other electrons), Landau postulated quasiparticles (QP) with charge *e* and spin $\frac{1}{2}$, which can be treated as nearly free electrons but carry a renormalized mass *m*^⋆^ that incorporates all interaction effects^1^. In his Fermi-liquid picture, the conductivity of metals scales with the QP lifetime *τ*, which increases asymptotically at low energy as the scattering phase space shrinks to zero^1^. Electron–electron interaction involves a quadratic energy dependence of the scattering rate *γ* = *τ*^−1^ on both temperature *T* and frequency *ω*^2–5^, expressed as:

1 $$\gamma (T,\omega)={\gamma }_{0}+B\left[{(p\pi {k}_{{\rm{B}}}/\hslash)}^{2}{T}^{2}+{\omega }^{2}\right].$$

**Fig. 1:**
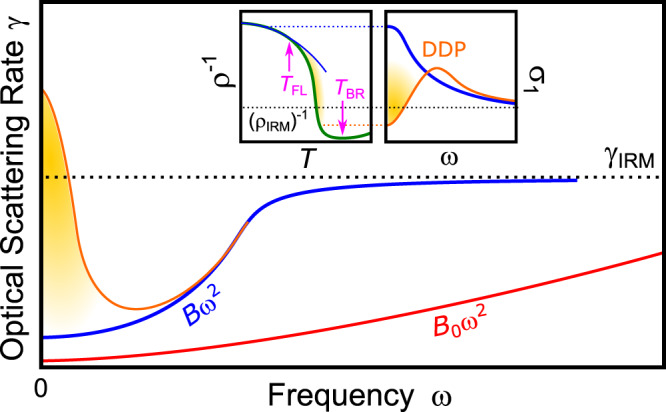
Scattering rate of correlated metals. In a Fermi liquid *γ*(*T*, *ω*) scales with *T*^2^ and *ω*^2^ due to the increase in scattering phase space (Eq. (1)). In common metals, electron–electron scattering is weak (red) and other dissipation processes dominate. The *ω*^2^ dependence prevails (blue) as electronic correlations yield *B* ≫ *B*_0_. While in good metals *γ*(*T*, *ω*) saturates at the Ioffe–Regel–Mott (IRM) limit^6,7^, dynamical localization^35^ can exceed this bound at low frequencies (orange). Insets: At *T* < *T*_FL_ the quasiparticle peak in the optical conductivity *σ*_1_ occurs at *ω* = 0. At *T*_FL_ < *T* < *T*_BR_, the resistivity (green, *ρ*^−1^ shown to compare with *σ*_1_) deviates from *ρ* ∝ *T*^2^ (blue) and increases beyond *ρ*_IRM_, which yields a drop of *σ*_1_ at low frequencies, forming a ‘displaced Drude peak’ (DDP) in a bad-metallic state.

Here *γ*_0_ stems from residual scattering processes at zero energy (e.g., impurities), and *p* is a numerical constant; the coefficient *B* controls the overall rate of variation with energy and increases with the effective mass *m*^⋆^. In most metals with large electronic bandwidth *W* the behavior described by Eq. (1) is not seen because of the small *m*^⋆^, so that the intrinsic contribution to scattering is negligible compared to other sources of dissipation (Fig. 1). Electronic correlations can strongly enhance the effective mass, *m*^⋆^/*m*_*b*_ ≫ 1 (in a local Fermi liquid the QP weight scales as $Z\propto {({m}^{* }/{m}_{b})}^{-1}$, where *m*_*b*_ is the band mass), making the energy-dependent terms of Eq. (1) the dominant contributions in the dc resistivity *ρ*(*T*) and optical conductivity *σ*_1_(*ω*).

While the quasiparticle concept has proven extremely powerful in describing good conductors, QPs become poorly defined in case of excessive scattering. In metals, the scattering rate is expected to saturate when the mean free path approaches the lattice spacing, known as Ioffe–Regel–Mott (IRM) limit^6,7^. However, this bound is often exceeded (*ρ* ≫ *ρ*_*IRM*_) in correlated, ‘Mott’ systems^8^. In view of the apparent breakdown of Boltzmann transport theory, it is controversially discussed whether charge transport in such *bad metals*^6,7^ is in any way related to QPs^9–11^ or whether entirely different excitations come into play^12^. By investigating the low-energy electrodynamics of a strongly correlated metal through comprehensive optical measurements, here we uncover the prominent role of QPs persisting into this anomalous transport regime, providing evidence for the former scenario. Our results also demonstrate the emergence of a ‘displaced Drude peak’ (DDP, see inset of Fig. 1) indicating incipient localization of QPs in a regime where their lifetime is already heavily reduced by strong electronic interactions.

## Results

**Fig. 2:**
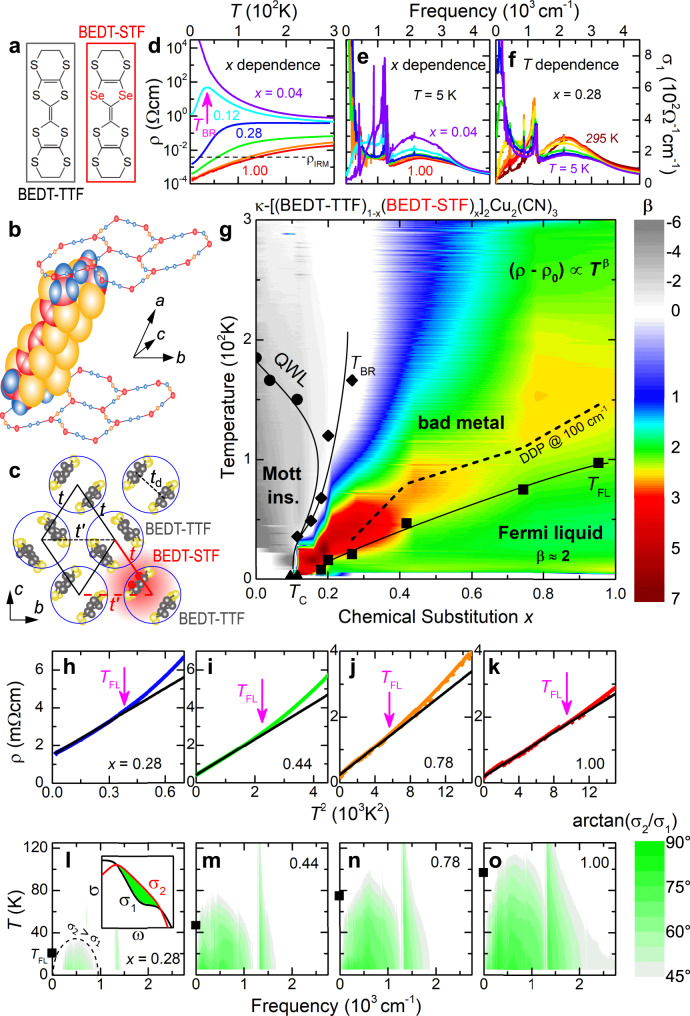
Mott transition to bad metal and Fermi liquid. **a**–**c** Introducing selenium-containing BEDT-STF molecules (red) in the layered triangular-lattice compound *κ*-(BEDT-TTF)_2_Cu_2_(CN)_3_ locally increases the transfer integrals. **d** This enhancement of electronic bandwidth by chemical substitution *x* yields a textbook-like Mott MIT in the resistivity of *κ*-STF_*x*_. **e**, **f** The optical conductivity reveals the formation of a correlated metallic state when increasing *x* and reducing *T*. **g** Consistent with theory (see Fig. 3 of ref. ^21^), the resistivity exponent $\beta ={\rm{d}}(\mathrm{ln}\,\{\rho -{\rho }_{0}\})/{\rm{d}}(\mathrm{ln}\,\{T\})$ shows large negative values in the Mott-insulating state (gray) below the quantum Widom line (QWL). On the metallic side, Fermi-liquid like *ρ* = *ρ*_0_ + *AT*^2^ at low temperatures (green) crosses over to a bad metal above *T*_FL_, featuring a rapid increase beyond *ρ*_IRM_. *β* ≫ 2 (orange-red) coincides with the displacement of the Drude peak from *ω* = 0 (DDP, dashed line). Metallic behavior is lost at *T*_BR_, where *ρ*(*T*) has a maximum. **h**–**k** The *AT*^2^ increase becomes steeper towards the MIT. **l**–**o** The phase angle determined from our optical experiments yields inductive semi-ellipses ($\arctan \{{\sigma }_{2}/{\sigma }_{1}\}\;> \;4{5}^{\circ }$ corresponds to *σ*_2_ > *σ*_1_) in *T* − *ω* domain as expected for a paradigmatic Fermi liquid^4,26^. The sharp feature occuring for all compounds at 1200 cm^−1^ is a vibration mode (see Supplementary Fig. 5).

We have chosen the molecular charge-transfer salts *κ*-[(BEDT-STF)_*x*_(BEDT-TTF)_1−*x*_]_2_Cu_2_(CN)_3_ (abbreviated *κ*-STF_*x*_), which constitute an ideal realization of the single-band Hubbard model on a half-filled triangular lattice. In the parent compound of the series (*x* = 0), strong on-site Coulomb interaction *U* = 2000 cm^−1^ (broad maximum of *σ*_1_(*ω*) in Fig. 2e, f) gives rise to a genuine Mott-insulating state^13,14^ with no magnetic order^15^ down to *T* = 0. Partial substitution of the organic donors by Se-containing BEDT-STF molecules with more extended orbitals^16^ increases the transfer integrals *t* ∝ *W* (Fig. 2a–c). As a result, the correlation strength *U*/*W* is progressively reduced with *x*, allowing us to tune the system through the “bandwidth-controlled” Mott metal-insulator transition (MIT), covering a wide range in *k*_B_*T*/*W* and *ℏω*/*W* within the parameter ranges accessible in our transport and optical experiments (see Supplementary Notes 2 and 3).

*ρ*(*T*) of *κ*-STF_*x*_ (Fig. 2d) reveals a textbook Mott MIT resembling the pressure evolution of *κ*-(BEDT-TTF)_2_Cu_2_(CN)_3_^17–19^, which turns metallic around 1.3 kbar. As *x* rises further, Fermi-liquid behavior *ρ*(*T*) = *ρ*_0_ + *AT*^2^ stabilizes below a characteristic *T*_FL_ that increases progressively with *x*, while $A\propto {({m}^{\star })}^{2}$ is reduced^20^ as correlations diminish (Fig. 2h–k). As common for half-filled Mott systems^10,21^, *ρ*(*T*) rises faster than *T*^2^ above *T*_FL_, seen by the effective temperature exponent *β* ≫ 2 in Fig. 2d, and exceeds *ρ*_IRM_ = *hd*/*e*^2^ = 4 mΩ cm (*h* = 2*πℏ* is Planck’s constant, *e* the elementary charge, and *d* = 16 Å the inter-layer spacing). We note that the bad metal formed here does not exhibit a linear-in-*T* resistivity that occurs in many other materials^12^. Instead, metallic behavior is completely lost when temperature exceeds the kinetic energy of QPs at the Brinkman–Rice scale *k*_B_*T*_BR_ ≃ *ZE*_F_^9,22^, and *ρ*(*T*) resembles a thermally activated semiconductor above the resistivity maximum^23,24^. Also in the optical conductivity we observe the transition from an insulator (d*σ*_1_/d*ω* > 0 at low frequencies) to a metal (d*σ*_1_/d*ω* ≤ 0), forming a QP peak at *ω* = 0 upon increasing *x* and lowering *T* (Fig. 2e, f). The phase diagram in Fig. 2g summarizes the characteristic crossovers in *κ*-STF_*x*_ (black symbols), also including the quantum Widom line (QWL)^13,21,25^ that separates the Mott insulator with a well-defined spectral gap from the incoherent semiconductor at elevated temperatures.

The low-energy response of Fermi liquids and the corresponding quadratic scaling laws are well explored theoretically^2–5^. A scattering rate *γ* ∝ *ω*^2^ implies an inductive response characterized by *σ*_2_ > *σ*_1_, where *σ*_1_ and *σ*_2_ are the real and imaginary part of the optical conductivity, respectively. This occurs in the so-called ‘thermal’ regime^4^, *ω* > *γ*, delimited by semi-elliptical regions in *T* − *ω* domain, as recently reported in Fe-based superconductors^26^. Our optical data on *κ*-STF_*x*_ indeed reveal inductive behavior, signaled by characteristic semi-ellipses with a phase angle $\arctan ({\sigma }_{2}/{\sigma }_{1})\;> \; 4{5}^{\circ }$ (Fig. 2l–o), and occurring at temperatures where *ρ* ∝ *T*^2^ is seen in dc transport (Fig. 2h–k), i.e., at *T* < *T*_FL_ (black squares). Concerning the *ω*^2^/*T*^2^ scaling in Eq. (1), Fermi-liquid theory predicts a ‘Gurzhi parameter’ *p* = 2 for the optical scattering rate *γ*(*T*, *ω*)^2^, a quantity that can be extracted from the optical conductivity via extended Drude analysis. Experimentally, values both in the range 1 ≤ *p* ≤2^26–29^ and *p* ≥2^30^ have been found for *γ*(*T*, *ω*) in few selected materials. While purely inelastic scattering among QPs yields *p* = 2, deviations towards *p* = 1 have been assigned to elastic scattering off quasi-static impurities (dopants, *f*-electrons), for instance^5^. It remains an open question how the *T* and *ω* dependences, and the value of *p*, develop as correlations advance towards the Mott MIT.

**Fig. 3:**
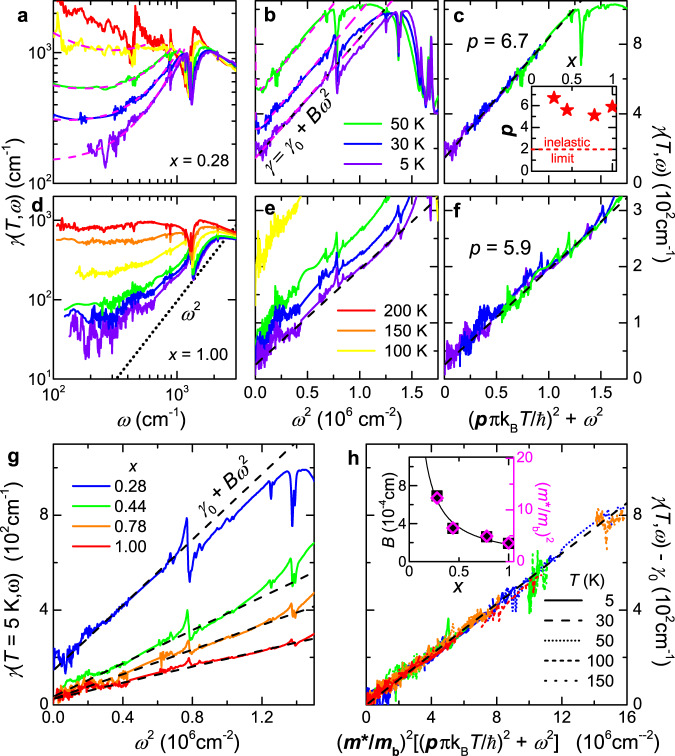
Fermi-liquid scaling of optical scattering rate obtained from extended Drude analysis. **a**, **d** *γ*(*T*, *ω*) acquires a pronounced frequency dependence at low *T*, here shown for *x* = 0.28 and 1.00. **b**, **e** *ω*^2^ behavior persists well above *T*_FL_; note the quadratic frequency scales. Dashed pink lines in **a**, **b** are fits to Eq. (2). **c**, **f** Curves recorded at different *T* collapse on a generalized quadratic energy scale (see Eq. (1)) for a specific Gurzhi parameter *p* > 2, as shown in the inset. **g** Comparing *γ*(*ω*) at 5 K for *x* ≥ 0.28 reveals that the slope of *Bω*^2^ increases towards the Mott MIT, similar to *AT*^2^ in dc transport (Fig. 2h–k). **h** Rescaling the energy dependence by ${({m}^{\star }/{m}_{b})}^{2}$ (see Supplementary Fig. 10) collapses all data on a universal scaling curve, which follows from $B\propto {({m}^{\star }/{m}_{b})}^{2}$. The 5, 30, and 50 K data are shown for all four substitutions (color code like in **g**); the scaling holds even for *T* ≥ 100 K for *x* ≥ 0.44. In panels **b**, **c**, **e**, **f**, **h** only the range below the vibrational features at 880 or 1200 cm^−1^ is considered.

In *κ*-STF_*x*_, the broadband response follows *γ* ∝ *ω*^2^ at low *T* (Fig. 3), as expected from Eq. (1), in all the compounds of the series (*x* = 0.28, 0.44, 0.78, and 1.00) that also show Fermi-liquid behavior in *ρ*(*T*). The pronounced dip visible in the spectra around 1200 cm^−1^ stems from a vibration mode with Fano-like shape in *σ*_1_(*ω*) and does not affect the relevant low-frequency behavior (see Fig. 2e, f and Supplementary Fig. 5). Analogue to the increase of the slope *A* in Fig. 2h–k, the *ω*^2^ variation of the scattering rate also becomes steeper as correlations gain strength (Fig. 3g), i.e., the coefficient *B* increases as *x* is reduced. In both cases, the quadratic energy dependence (and any d*γ*/d*ω* > 0) appears only below *γ*_IRM_ = 1000 cm^−1^.

The stringent prediction Eq. (1) can be directly verified by adding the *T*^2^- and *ω*^2^-dependences of *γ*(*T*, *ω*) to a common energy scale. In Fig. 3c, f, the curves at different *T* do fall on top of each other upon scaling via a Gurzhi parameter *p* = 6 ± 1 for all *κ*-STF_*x*_ (see inset of panel c and Supplementary Fig. 11e–h). Even more striking, multiplying the energy scale by ${\left({m}^{\star }/{m}_{b}\right)}^{2}$ collapses the data of all four substitutions on one universal line (Fig. 3h). This manifestation of the Kadowaki–Woods relation^20^, $B\propto {({m}^{\star })}^{2}$ (see inset), rules out any relevance of spinons near the Mott MIT^19,31^, in accord with dynamical mean-field theory (DMFT) results^32^. All in all, the observed scaling provides compelling evidence for the applicability of Landau’s Fermi-liquid concept, in agreement with previous studies on unconventional superconductors^26,28,29^. The Gurzhi parameter significantly exceeds the inelastic limit (*p* = 2)^5^, indicating quasi-elastic backscattering processes (see Eqs. (2) and (3) below).

**Fig. 4:**
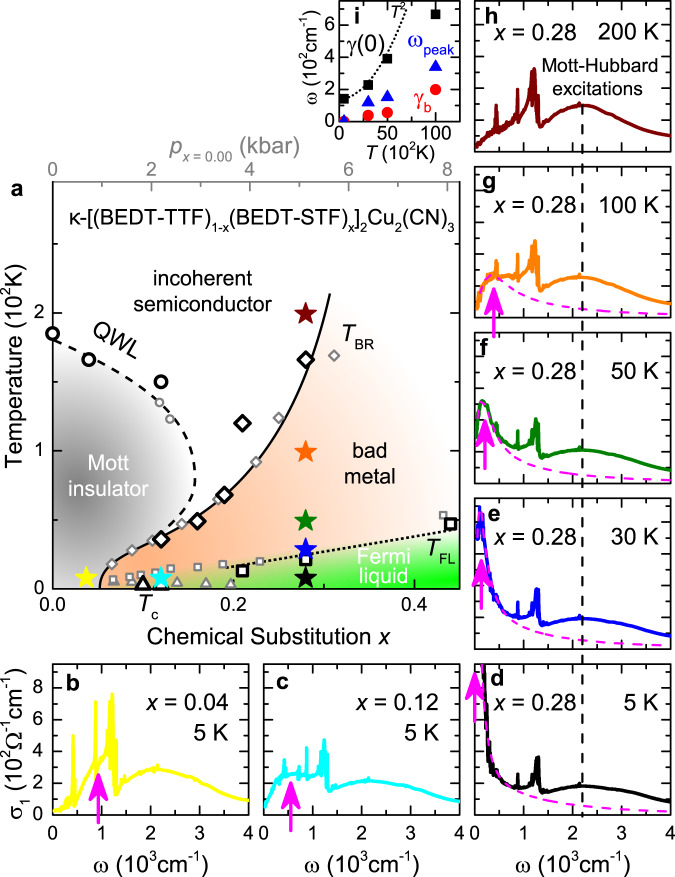
Displaced Drude peak linked to bad metal. **a** Chemical substitution *x* (black symbols) and physical pressure *p* (gray, see Supplementary Fig. 2) have the same effect on *κ*-(BEDT-TTF)_2_Cu_2_(CN)_3_^24^. **b**–**h** Evolution of *σ*_1_(*T*, *ω*, *x*) through the phase diagram; stars with respective color indicate the position in **a**. Entering the bad-metallic phase for *T* > *T*_FL_ shifts the Drude peak away from *ω* = 0. The maximum broadens and hardens with *T*, until it dissolves at *T*_BR_. Approaching the insulator (*x* = 0.28 → 0.04) at low *T*, the QP feature transforms into finite-frequency metallic fluctuations within the Mott gap^13^. **i** Fit parameters (Eq. (2)) for dashed pink lines in **d**–**g** and Fig. 3a, b. Dotted line indicates $\gamma (0)={\gamma }_{0}+B{(p\pi {k}_{{\rm{B}}}T/\hslash)}^{2}$, with *B* and *p* from Fig. 3.

Having analyzed the QP properties and their dependence on electronic correlations, we now want to evaluate how they behave when scattering increases as we cross over from the Fermi liquid into a bad metal. Figure 4 displays *σ*_1_(*ω*) at distinct positions in the *T*–*x* phase diagram (stars in panel a); note the similarity between *κ*-STF_*x*_ (black symbols) and *κ*-(BEDT-TTF)_2_Cu_2_(CN)_3_ subject to pressure tuning (gray). For all *x* ≥ 0.28 and *T* < *T*_FL_, the optical spectra feature a Drude-like peak centered at *ω* = 0, representing the QP response, together with a broad absorption centered at *U* = 2000 cm^−1^ originating from electronic transitions between the Hubbard bands^13^, as shown in Fig. 4d (see also Fig. 2e, f and Supplementary Fig. 6). While the high-energy features show only weak dependence on *x* and *T*, a marked shift of spectral weight takes place within the low-frequency region (see Supplementary Note 3). The fingerprints of mobile carriers evolve upon moving away from the Fermi-liquid regime by either changing *x* (Fig. 4b–d) or increasing *T* (d-h), until they completely disappear both in the Mott insulator (panel b) and in the incoherent semiconductor (panel h, *T* > *T*_BR_ = 166 K at *x* = 0.28).

Closer scrutiny reveals that this gradual evolution of the low-frequency absorption is accompanied by the appearance of a dip at *ω* = 0, which occurs at *T* ≥ *T*_FL_; this is also where the resistivity becomes anomalous, deviating from *ρ* ∝ *T*^2^. The QP response then evolves into a finite-frequency peak, that steadily shifts to higher *ω* and broadens with increasing *T*/reducing *x* (arrows in Fig. 4e–g and triangles in panel i). Such a displaced Drude peak eventually dissolves into the Hubbard band at *T* ~ *T*_BR_. From contour plots of *σ*_1_(*T*, *ω*) in Supplementary Fig. 7 we can estimate the *T*–*ω* trajectory of the DDP above *T*_FL_ for the different substitutions: a peak frequency around 100 cm^−1^ (dashed line in Fig. 2g) coincides with the steepest increase of the resistivity, i.e., the largest values of the exponent *β* > 2.

The emergence and fading of the DDP at *T*_*FL*_ and *T*_BR_, respectively, indicate that the observed behavior is tightly linked to the bad-metal response in the resistivity, tracking the changes experienced by the QPs as the Fermi liquid degrades. This physical picture is reminiscent of the recently introduced concept^9–11^ of ‘resilient’ QPs, which persist beyond the nominal Fermi-liquid regime, but with modified (e.g., *T*-dependent) QP parameters. Note that the DDP phenomenon observed here, that is not predicted by current theoretical descriptions of Mott systems^9,33^, also impacts charge transport itself: for example, the values of *σ*_1_ and *γ* seen at finite frequency in our optical experiments, which yield correlation strengths *U*/*W* = 1.3 for *x* ≥ 0.28 (see Supplementary Fig. 6), are compatible with those computed by DMFT^33^, but the measured dc resistivity increases way beyond the theory values — a natural consequence of the drop of *σ*_1_ at low frequencies upon DDP formation.

Building on the considerations above, we now show that our experimental observations can be explained by an incipient localization of the carriers in the bad metal, caused by non-local, coherent backscattering corrections to semi-classical transport^34,35^. We note that related ideas have been invoked to explain the bad metallicity and DDP observed in liquid metals^34^ and various correlated systems, including organics^36^, cuprates^37,38^, and other oxides^39^, but no systematic quantitative investigations have been provided to date.

In order to describe the experimental observations, we now introduce a model that assigns the modifications of the Drude peak to backscattering processes^34,35,40,41^:

2 $$\sigma (\omega)=\frac{{\epsilon }_{0}{\omega }_{p}^{2}}{\gamma -{\gamma }_{b}}\left[\frac{\gamma }{\gamma -i\omega }-\frac{{\gamma }_{b}}{{\gamma }_{b}-i\omega }\right].$$

Here the first term between brackets represents the standard metallic response with the energy-dependent *γ* from Eq. (1), where *ω*_*p*_ is the plasma frequency. The second term represents the leading finite-frequency correction beyond semiclassical transport, caused by additional elastic or quasi-elastic processes. Its sign is opposite to that of the semiclassical Drude response, leading to a dip-peak structure in *σ*_1_(*ω*) as illustrated in the inset of Fig. 1. The resulting peak frequency, ${\omega }_{peak}\simeq \sqrt{\gamma (0){\gamma }_{b}}$, gives a direct measure of the backscattering rate *γ*_*b*_. Physically, the “localization” corrections embodied in Eq. (2) represent non-local interference processes, which can be viewed as finite-frequency precursors of a disorder-induced bound-state formation.

We have used Eq. (2) to fit the finite-frequency spectra at the substitution *x* = 0.28, where the DDP is most clearly identified in the experiment. The Fermi-liquid response has been extracted from Fig. 3, setting a constant *B* = 6.7 × 10^−4^ cm at all temperatures up to *T* = 100 K. Importantly, ${\omega }_{p}^{2}$ is also kept constant, compatible with the fact that the spectral weight associated with the QPs is conserved from the Fermi liquid to the bad-metallic region. The fits accurately describe the experimental data, as demonstrated by magenta lines for *σ*_1_(*T*, *ω*) in Fig. 4d–g and for *γ*(*T*, *ω*) in Fig. 3a, b. Similar to the direct determination from the extended Drude analysis, the extracted *γ*(0) shows an initial *T*^2^ dependence which is lost at *T* ≥ *T*_FL_, as illustrated in Fig. 4i. The parameter *γ*_*b*_ ≪ *γ*(0) shows a similar trend.

To get further microscopic insight, we isolate explicitly the anomalous scattering contributions by writing

3 $$\delta \gamma (\omega ,T)=\gamma (\omega ,T)-{\gamma }_{{\rm{FL,2}}}(\omega ,T),$$

where *γ*(*ω*, *T*) is the measured scattering rate, which has the general form Eq. (1), and *γ*_FL,2_ is the strict Fermi-liquid prediction, i.e., Eq. (1) with *p* = 2. Direct comparison with Eq. (1) yields $\delta \gamma (\omega ,T)=B({p}^{2}-{2}^{2}){(\pi {k}_{B}T/\hslash)}^{2}$, from which the following conclusions can be drawn. First, the fact that we find a frequency-independent correction directly confirms the assumed (quasi)static nature of the anomalous scatterers. Second, the fact that *p* = 6 ± 1 is almost constant for all substitutions (Fig. 3c, inset) means that the strength of *δγ* (in particular its variation with *x*) is governed by the QP scale embodied in the parameter *B*, i.e., $\delta \gamma \sim {(\frac{{m}^{\star }}{{m}_{b}})}^{2} \sim {Z}^{-2}$. This observation stresses the key role of strong correlation effects in the vicinity of the Mott point. Third, *p* ≫ 2 implies that the anomalous contribution *δγ* is dominant over the inelastic term, which consistently ensures that the corresponding localization effects are robust against the dephasing effects originating from inelastic QP scattering: whenever observed, the peak frequency *ω*_*peak*_ is much larger than the calculated dephasing term, ~ *Bω*^2^.

## Discussion

The *κ*-[(BEDT-STF)_*x*_(BEDT-TTF)_1−*x*_]_2_Cu_2_(CN)_3_ series studied here realizes a continuous tuning through the genuine Mott MIT near *T* → 0 that was previously not accessible by experiments applying physical pressure. Our systematic investigation of the electron liquid from the weakly interacting limit to the Mott insulator establishes Landau’s QPs as the relevant low-energy excitations throughout the metallic phase. While demonstrating the universality of Landau’s QP picture, the foregoing analysis also reveals an enhanced elastic scattering channel that fundamentally alters the QP properties in these materials. This is best visible within the bad-metallic regime, where it conspires with electronic correlations in causing a progressive shift of the Drude peak to finite frequencies, indicative of dynamical localization of the QPs. Our analysis also suggests that the same elastic processes may already set in within the Fermi-liquid regime, causing deviations from the predicted *ω*^2^/*T*^2^ scaling laws of QP relaxation. These conclusions are largely based on a straightforward analysis of experimental data by a general theoretical model describing the optical response of charge carriers in the presence of incipient localization. We now discuss possible scenarios to elucidate the possible microscopic origins. The key feature that requires explanation is the pronounced elastic scattering near the Mott point.

One firmly established example leading to DDP behavior and anomalously high resistivities is the “transient localization” phenomenon found in crystalline organic semiconductors^42^. There, soft lattice fluctuations provide a strong source of quasi-elastic randomness at room temperature, causing coherent backscattering at low frequency and DDPs^41,43^. In the present *κ*-STF_*x*_ compounds the Debye temperature for the relevant inter-molecular phonons, *T*_*D*_ ~ 30*K*, is similar to that of organic semiconductors, compatible with transient localization at high *T*. However, this picture is difficult to reconcile with the observed DDP at very low *T* close to the MIT that exhibits strong substitution dependence, indicating instead a clear connection with the Mott phenomenon. Similar caveats would apply if lattice fluctuations were replaced by other soft bosons unrelated to the Mott MIT, such as charge/magnetic collective modes. While the latter can also give rise to finite-frequency absorption peaks, our clear assignment of the DDP to metallic QP rules out such a situation in the present case^44,45^.

An alternative possibility, that could reconcile the different experimental observations, is the physical picture of weakly disordered Fermi liquids^46,47^, motivated by the unavoidable structural disorder that accompanies chemical substitution^16^. Although a complete theory for such a situation is still not available, existing studies^47^ show that disorder directly affects the Fermi liquid, making its coherence scale *T*_BR_ spatially inhomogeneous with a broad distribution of local QP weights. In this case, one expects local regions with low *T*_FL_ to ‘drop out’ from the Fermi liquid and thus act essentially as vacancies — dramatically increasing the elastic scattering as temperature is raised. While providing a plausible physical picture for *p* > 2, this scenario would also be consistent with the observed scaling of *γ* with ${(\frac{{m}^{\star }}{{m}_{b}})}^{2}$ upon approaching the Mott point, reflecting the gradual build-up of correlations in the disordered Fermi liquid.

Finally, we argue that long-range Coulomb interactions, that are usually neglected in theoretical treatments of correlated electron systems, could actually play a key role both in the present compounds as well as in other bad metals where DDPs have been reported^45^. The ability of non-local interactions in providing an effective disordered medium for lattice electrons has been recognized recently^48–51^, with direct consequences on bad-metallic behavior^52^. The additional scattering channel associated with long-range potentials could well be amplified at the approach of the Mott transition, due to both reduced screening and collective slowing down of the resulting randomness, possibly causing DDP behavior as observed here.

Since the gradual demise of quasiparticles is a general phenomenon in poor conductors, displaced Drude peaks likely occur in many of them^45^. In light of the present experiments, studying the interplay between electronic correlations and (self-induced) randomness appears to be a very promising route for understanding how good metals turn bad.

## Methods

### Experimental

Plate-like single crystals of *κ*-[(BEDT-STF)_*x*_(BEDT-TTF)_1−*x*_]_2_Cu_2_(CN)_3_ were grown electrochemically^16^ with a typical size of 1 × 1 × 0.3 mm^3^; here BEDT-TTF stands for bis(ethylenedithio)tetrathiafulvalene and BEDT-STF denotes the partial substitution by selenium according to Fig. 2a. The composition of 0 ≤ *x* ≤ 1 was determined by energy-dispersive X-ray spectroscopy^16^. The dc resistivity was recorded by standard four-point measurements; superconductivity was probed by magnetic susceptibility studies of polycrystalline samples using a commercial SQUID and magnetoresistance measurements on single crystals. We performed complementary pressure-dependent transport experiments of the parent compound (*x* = 0), shown in Supplementary Fig. 2b, providing the gray data points in Fig. 4a. Since the compounds are isostructural, they retain the highly frustrated triangular lattice and do not exhibit magnetic order down to lowest temperatures; a hallmark of the spin-liquid state. Using Fourier-transform infrared spectroscopy, the optical reflectivity at normal incidence was measured in the frequency range from 50 to 20000 cm^−1^ from *T* = 5 K up to room temperature; here also the visible and ultraviolet regimes were covered up to 47,600 cm^−1^ by a Woollam ellipsometer. The complex optical conductivity $\hat{\sigma }(\omega)={\sigma }_{1}(\omega)+{\rm{i}}{\sigma }_{2}(\omega)$ is obtained via the Kramers–Kronig relations using standard extrapolations. Since the optical properties of both crystal axes provide similar information, we focus on the spectra acquired for the polarization along the crystallographic *c*-axis.

### Extended drude analysis

The frequency-dependent scattering rate and effective mass are calculated via the extended Drude model^53,54^

4 $$\gamma (\omega)={\epsilon }_{0}{\omega }_{p}^{2}\ {\rm{Re}}\left\{{[\hat{\sigma }(\omega)]}^{-1}\right\}$$

5 $$\frac{{m}^{\star }(\omega)}{{m}_{b}}=\frac{{\epsilon }_{0}{\omega }_{p}^{2}}{\omega }\ {\rm{Im}}\left\{-{[\hat{\sigma }(\omega)]}^{-1}\right\},$$

where ${\omega }_{p}=\sqrt{N{e}^{2}/{\epsilon }_{0}{m}_{b}}$ is the plasma frequency, comprising the charge-carrier density *N* and band mass *m*_*b*_; *ϵ*_0_ is the permittivity of vacuum and *e* the elementary charge. *ω*_*p*_ is determined from the maximum of the dielectric loss function around 4000 cm^−1^ (see Supplementary Fig. 9).

## Supplementary information

Supplementary Information

## Data Availability

The authors declare that the data supporting the findings of this study are available within the paper and its Supplementary Information. Further information can be provided by A.P., M.D., or S.F.
