# Supplementary material for: Rise and fall of Landau’s quasiparticles while approaching the Mott transition
Source: Nat Commun. 2021 Mar 10;12:1571. doi: 10.1038/s41467-021-21741-z (PMC7977040; doi:10.1038/s41467-021-21741-z)
Supplement: Supplementary file 1 — Supplementary Information [file 41467_2021_21741_MOESM1_ESM.pdf]

Supplementary Information

Rise and Fall of Landau's Quasiparticles While Approaching the Mott Transition

A. Pustogow⋆,[1,2] Y. Saito,[1,3] A. Löhle,[1] M. Sanz Alonso,[1] A. Kawamoto,[3] V. Dobrosavljević,[4] M. Dressel,[1] and S. Fratini[5]

[1] *1. Physikalisches Institut, Universität Stuttgart, 70569 Stuttgart, Germany*

[2] *Department of Physics and Astronomy, UCLA, Los Angeles, California 90095, USA*

[3] *Department of Physics, Graduate School of Science, Hokkaido University, Sapporo 060-0810, Japan*

[4] *Department of Physics and National High Magnetic Field Laboratory, Florida State University, Tallahassee, Florida 32306, USA*

[5] *Institut Néel - CNRS and Université Grenoble Alpes, 38042 Grenoble Cedex 9, France*

(Dated: January 20, 2021)

CONTENTS

Supplementary Note 1: Crystal Structure 1

Supplementary Note 2: dc Resistivity 2

Supplementary Note 3: Optical Spectroscopy 4

Supplementary Note 4: Extended Drude Analysis 8

Supplementary References 10

SUPPLEMENTARY NOTE 1: CRYSTAL STRUCTURE

Single crystals of κ-[(BEDT-STF)$_x$(BEDT-TTF)$_{1-x}$]$_2$Cu$_2$(CN)$_3$ with the stoichiometries $x = 0$, 0.04, 0.1, 0.12, 0.16, 0.19, 0.21, 0.25, 0.28, 0.44, 0.78 and 1.00 were prepared by standard electrochemical oxidation [1]. BEDT-TTF stands for bis(ethylenedithio)tetrathiafulvalene and BEDT-STF for bis-(ethylenedithio)diselenium-dithiafulvalene. While BEDT-TTF molecules were purchased from Sigma-Aldrich, the synthesis of BEDT-STF and the crystal growth were carried out at Hokkaido University in Sapporo. The two types of molecules are displayed in Supplementary Fig. 1a. In order to create different substitutions in the alloy series, the amount of donor molecules was preselected; the actual value x was determined *a posteriori* by energy-dispersive x-ray spectroscopy for each batch: we compared the intensity of S atoms to that of Se atoms, using κ-(BEDT-TTF)$_2$Cu$_2$(CN)$_3$ as a reference. More information on crystal growth can be found in Supplementary Ref. 2.

⋆Present Address: Institute of Solid State Physics, Vienna University of Technology, 1040 Vienna, Austria.

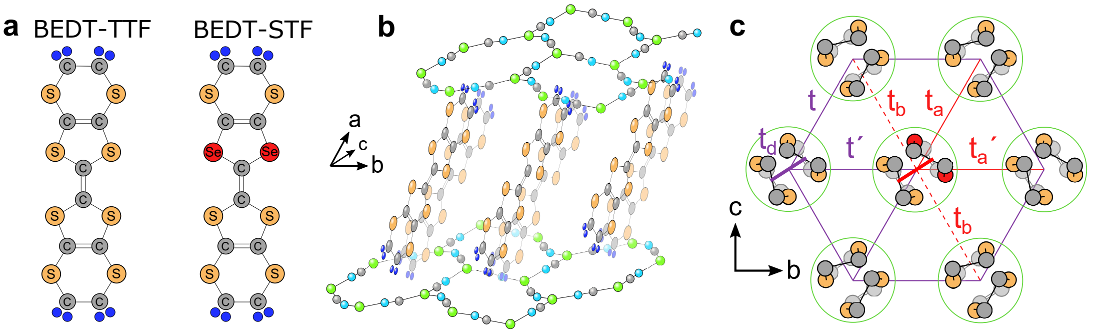

Supplementary Figure 1. **Crystal structure. a,** Organic donor molecules BEDT-TTF and BEDT-STF. For the latter, two of the inner sulfur atoms are substituted by selenium. **b,** The crystal structure consists of layers of the donor molecules along the bc-plane that are separated by the polymeric $Cu_2(CN)_3$ anion network. **c,** Within the layers, the BEDT-TTF (BEDT-STF) dimers form a slightly anisotropic triangular arrangement with transfer integrals $t'/t = 0.83$ [3], i.e. the lattice is subject to significant geometrical frustration. Substitution of BEDT-TTF by BEDT-STF enlarges the transfer integrals (red) due to the extended orbitals of the selenium-containing BEDT-STF molecules. Note, also the intra-dimer transfer integrals, $t_d \gg t$,t', are enhanced by substitution.

The BEDT-TTF (resp. BEDT-STF) molecules form dimers because the intra-dimer transfer integral t_d is significantly larger than the inter-dimer wave function overlaps t and t' (Supplementary Fig. 1c). Per dimer one electron is donated to the $[Cu_2(CN)_3]^-_\infty$ anion sheets that separate the donor layers as shown in Supplementary Fig. 1b. Within the bc plane the dimers constitute a slightly anisotropic triangular lattice that is close to ideal geometrical frustration, $t'/t = 0.83$ [3] as illustrated in Supplementary Fig. 1c. The system is subject to strong electronic correlations as, overall, each dimer carries one hole with spin 1/2: on-site Coulomb repulsion among the charges realizes a Mott insulator at half filling [4]. In the alloys the BEDT-TTF molecules are randomly substituted by BEDT-STF, which mainly leads to an increase of the electronic bandwidth. It may also give rise to disorder in the transfer integrals, similar to chemical substitution or doping in other materials.

SUPPLEMENTARY NOTE 2: DC RESISTIVITY

Standard four-point dc transport experiments were carried out for the alloy series κ-$[(\text{BEDT-STF})_x(\text{BEDT-TTF})_{1-x}]_2Cu_2(CN)_3$ in the temperature range from $T = 300$ K down to 2 K. Supplementary Fig. 2a shows the complete set of resistivity curves $\rho(T)$ normalized to 300 K that were used to construct the phase diagrams in Fig. 2d and Fig. 4a. Prior to performing the logarithmic derivative, the residual resistivity ρ_0 in the limit $T \to 0$ has to be subtracted in order to yield the power-law exponent $\beta = \mathrm{d}(\ln\{\rho - \rho_0\})/\mathrm{d}(\ln\{T\})$. While the Fermi-liquid temperature T_{FL} is reached when the resistivity deviates from $\rho(T) = \rho_0 + AT^2$, the Brinkman-Rice temperature T_{BR} is defined when $\rho(T)$ goes through a maximum; both temperatures are indicated in Fig. 2. In Supplementary Fig. 3a,b we illustrate how the quantum Widom line is determined: the steepest slope in the Arrhenius plot corresponds to the maximum in $\mathrm{d}(\ln\{\rho\})/\mathrm{d}(1/T)$; this is the distinctive criterion for T_{QWL} (cf. Fig. S2 in Supplementary Ref. 9). As seen in Supplementary Fig. 2a, a Mott-insulating behavior occurs only for $x \leq 0.12$. The larger substitutions ($x \geq 0.12$) behave metallic below T_{BR}; this corresponds to a sign change in $\mathrm{d}(\ln\{\rho\})/\mathrm{d}(1/T)$ as seen in Supplementary Fig. 3b,c. Furthermore for κ-$[(\text{BEDT-STF})_x(\text{BEDT-TTF})_{1-x}]_2Cu_2(CN)_3$ with $x = 0.10$ and 0.12 we find that around 2.5 - 3 K superconductivity sets in at ambient pressure.

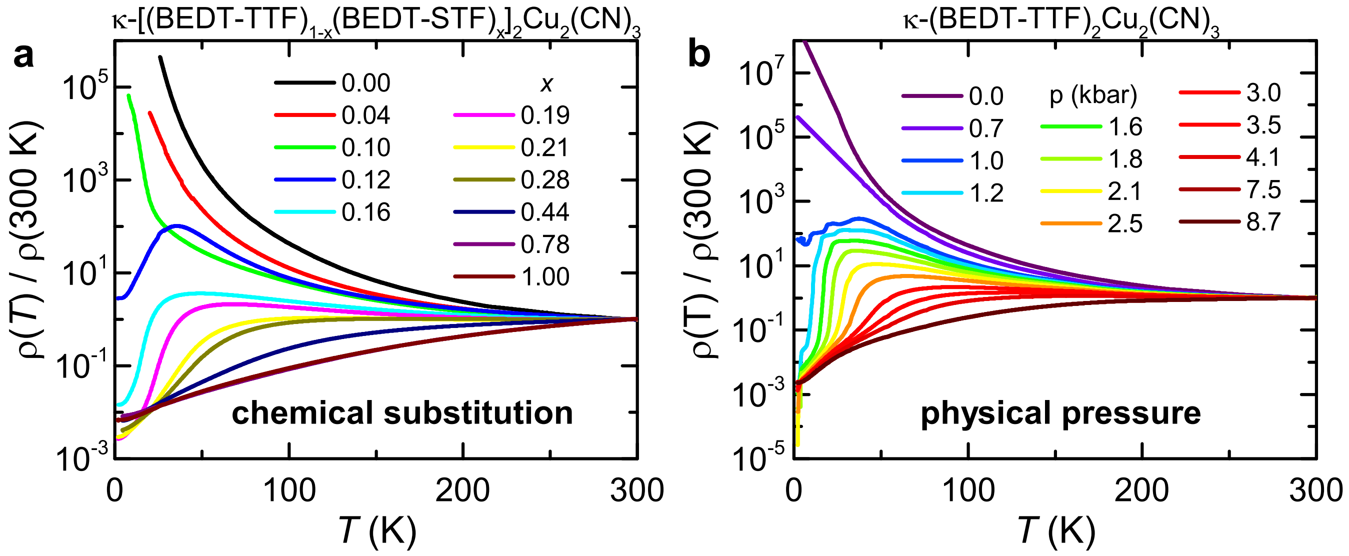

Supplementary Figure 2. **Substitutional and pressure evolution of the dc resistivity. a,** The temperature dependence of the dc resistivity (normalized to $T = 295$ K) is plotted for the series κ-[(BEDT-STF)$_x$(BEDT-TTF)$_{1-x}$]$_2$Cu$_2$(CN)$_3$ [5]. **b,** Temperature-dependent dc resistivity was measured on κ-(BEDT-TTF)$_2$Cu$_2$(CN)$_3$ for increasing hydrostatic pressure. The resistivity $\rho(T)$ is successively reduced with higher pressure: metallic behavior and superconductivity are observed at low temperatures, in agreement with literature [6–8]. The pressure values indicated here correspond to the lowest temperature. During cooling the pressure is reduced due to thermal contraction and freezing of the pressure-transmitting medium (Daphne oil). *In-situ* calibration allows us to determine the actual pressure at each temperature, which is taken into account in Fig. 3a.

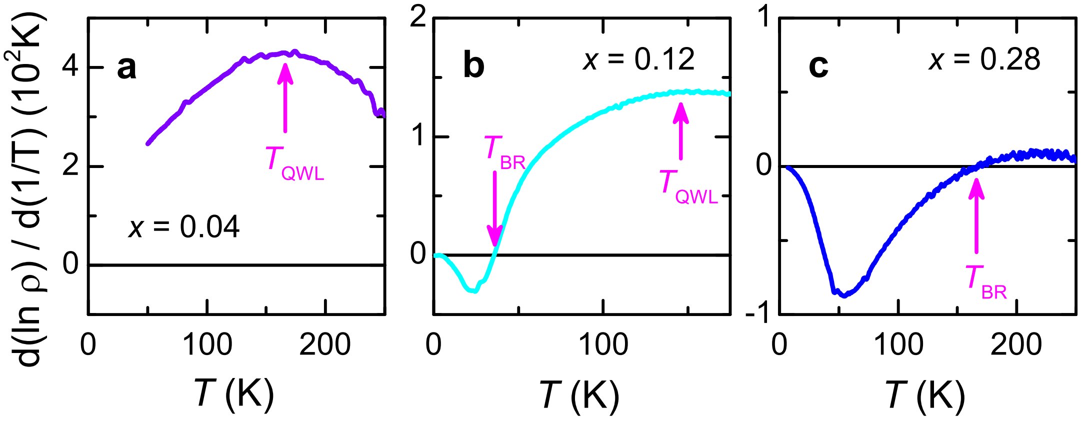

Supplementary Figure 3. **Transport gap defines quantum Widom line. a,b,** For those compounds that show Mott-insulating behavior, $x = 0.04$ and $x = 0.12$, a maximum in the logarithmic derivative indicates the temperature when $\rho(T)$ crosses the quantum Widom line. **b,c,** The change of sign defines the Brinkman-Rice temperature $T_{\rm BR}$ as the onset of metallic transport ($d(\ln\rho)/d(1/T) < 0$).

In addition to ambient-pressure dc transport experiments on the κ-[(BEDT-STF)$_x$(BEDT-TTF)$_{1-x}$]$_2$Cu$_2$(CN)$_3$ alloy series, we performed complementary pressure-dependent resistivity measurements on the parent compound ($x = 0$); the results are displayed in Supplementary Fig. 2b. Here the Mott insulator-metal transition occurs slightly above 1 kbar and is accompanied by superconductivity (onset at $T_{\rm c,max} \approx 5$ K), in agreement with previous work [6–8]. The resulting values for $T_{\rm QWL}$, $T_{\rm FL}$, $T_{\rm BR}$ and $T_{\rm c}$ are included in Fig. 4a; they line up well with the transition and crossover temperatures obtained via chemical substitution.

SUPPLEMENTARY NOTE 3: OPTICAL SPECTROSCOPY

Optical spectroscopic experiments were performed on the substitution series κ-[(BEDT-STF)$_x$(BEDT-TTF)$_{1-x}$]$_2$-Cu$_2$(CN)$_3$ covering a frequency range of 50–20000 cm^{-1} and for temperatures from 5 K up to 295 K. Due to the small in-plane anisotropy of the transfer integrals (Supplementary Fig. 1c), the dc resistivity differs by a factor 2 between the b and c directions [10]. In Supplementary Fig. 4 we compare the optical spectra of the two crystal axes. While for both polarizations the absorption exhibits a peak around 2500 cm^{-1} and 2000 cm^{-1}, respectively, along b-direction one can distinguish another feature above 3000 cm^{-1} which corresponds to intra-dimer excitations that do not contribute to charge transport [11–14]. Apart from minor quantitative differences, metallic and insulating behavior expresses for both in-plane axes as exemplarily shown here for the substitutions $x = 0.04$ and 0.78 in Supplementary Fig. 4. For the determination of U and W from the Mott-Hubbard transitions, we focused on the optical spectra acquired for $E \parallel c$.

In addition, a strong vibrational mode occurs around 1400 cm^{-1} related to intramolecular vibrations mainly involving C=C. Due to electron-molecular vibrational coupling, this feature can become rather intense and acquire an asymmetric Fano shape, in particular for in the insulating regime [15]. Although these vibrational features might appear disturbing, we refrain from subtracting them off the spectra in an arbitrary fashion; instead we present raw data whenever possible.

Supplementary Fig. 5 displays the full set of optical data ($x = 0.04, 0.12, 0.28, 0.44, 0.78, 1.00$; 5 K $< T <$ 295 K) of the alloying series discussed in the present work. Similar to the parent compound [16, 17], the substitution $x = 0.04$ exhibits an insulating-like optical conductivity with $\sigma_1(\omega \to 0) \to 0$ and $d\sigma_1/d\omega > 0$ below the Hubbard transitions at all temperatures. Also for $x = 0.12$ and 0.28, the high-temperature spectra ($T > T_{\mathrm{BR}}$) reveal insulating, or rather semiconducting properties – at finite temperature there is always a non-zero offset to σ_1 at $\omega = 0$ due to thermal

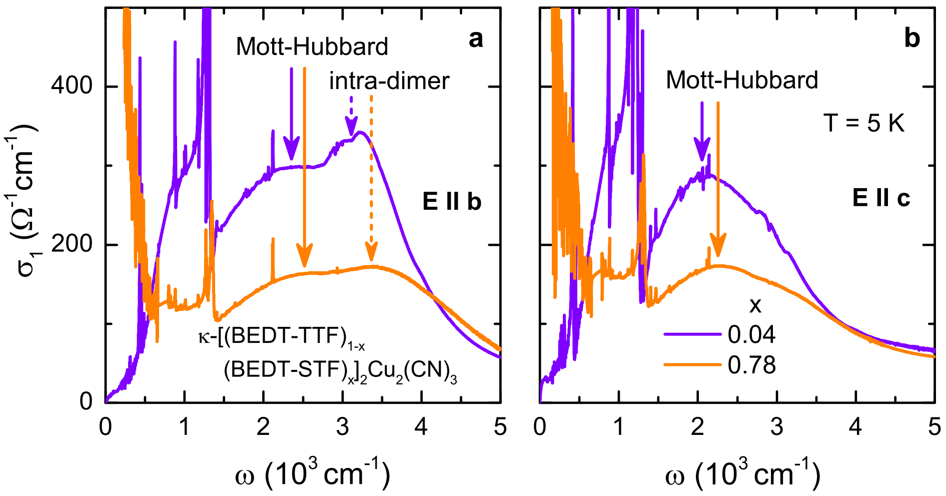

Supplementary Figure 4. **In-plane anisotropy.** The anisotropy of the in-plane electrodynamic response of κ-[(BEDT-STF)$_x$(BEDT-TTF)$_{1-x}$]$_2$Cu$_2$(CN)$_3$ between (a) $E \parallel b$ and (b) $E \parallel c$ is exemplified for $x = 0.04$ and 0.78 at $T = 5$ K. The main contribution along both crystal axes is due to Mott-Hubbard excitations between the dimers (solid arrows) [11–14]. Overall, the Coulomb repulsion U, associated with the band maximum, and the corresponding electronic correlation strength U/W are slightly larger for $E \parallel b$ [9]. Local intra-dimer excitations show up above 3000 cm^{-1} (dashed lines) and are more pronounced along the b-direction as compared to $E \parallel c$. Both electronic contributions shift to higher energy with increasing STF content x.

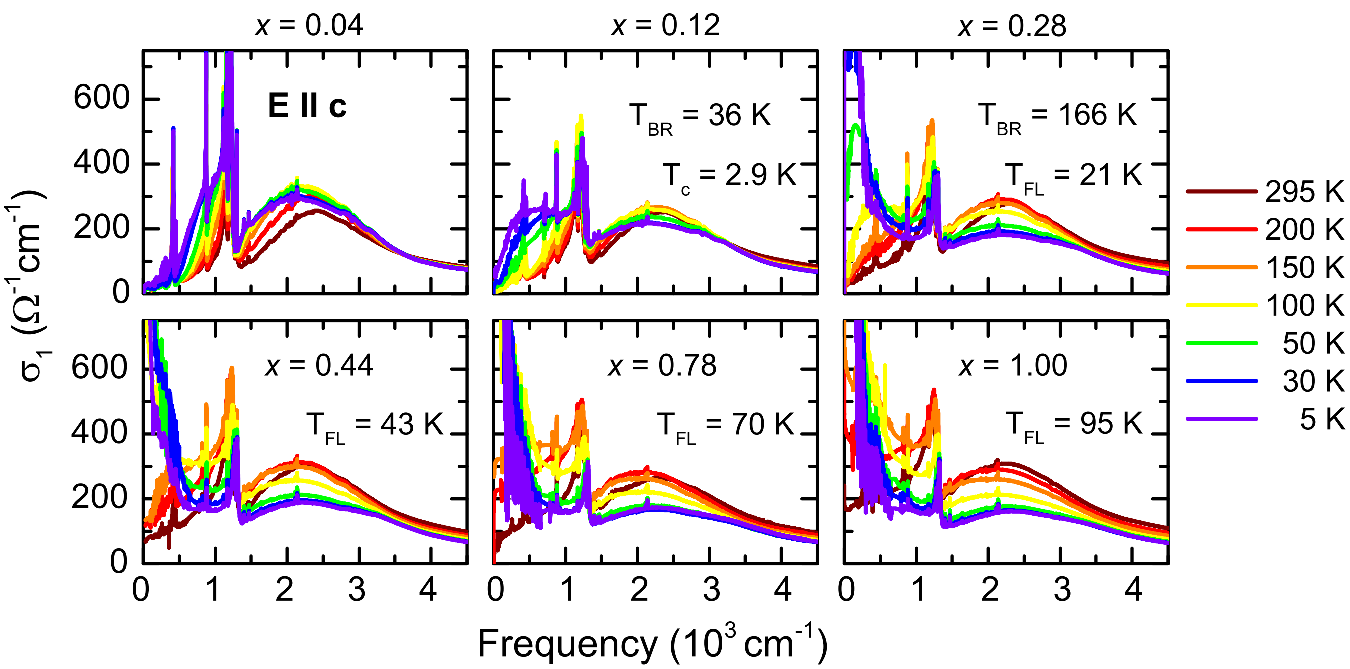

Supplementary Figure 5. **Temperature-dependent optical conductivity for all substitutions.** The optical conductivity of κ-[(BEDT-STF)$_x$(BEDT-TTF)$_{1-x}$]$_2$Cu$_2$(CN)$_3$ was determined in reflection experiments from room temperature down to $T =$ 5 K. As substitution $x = 0.04, 0.12, 0.28, 0.44, 0.78, 1.00$ increases, spectral weight successively accumulates at low frequencies coincident with the onset of metallic behavior below T_{BR}. A Drude-like peak forms at zero frequency for $T < T_{\mathrm{FL}}$.

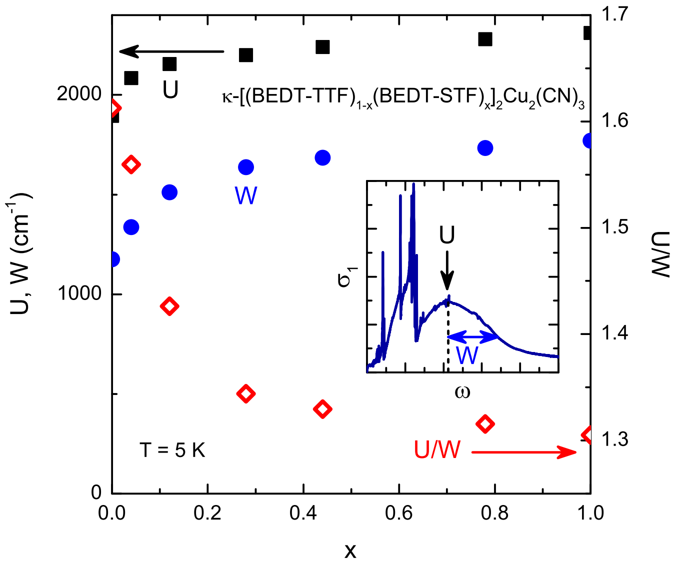

Supplementary Figure 6. **Band parameters U, W and U/W of κ-STF$_x$.** The electronic bandwidth W and on-site Coulomb repulsion U were determined from the half-width at half maximum and the peak position, respectively. The increase of W is more pronounced for $x \to 1$ leading to an overall reduction of the correlation strength U/W (red symbols, right scale).

excitations across the gap. Below T_{BR}, however, a significant amount of the spectral weight accumulates at low frequencies. Eventually, a Drude-like peak is established at $\omega = 0$ for $T < T_{\mathrm{FL}}$ in all substitutions $x \geq 0.28$ that contain Fermi-liquid properties in dc transport.

In Supplementary Fig. 6 we present the band parameters: the Coulomb repulsion U and the electronic bandwidth

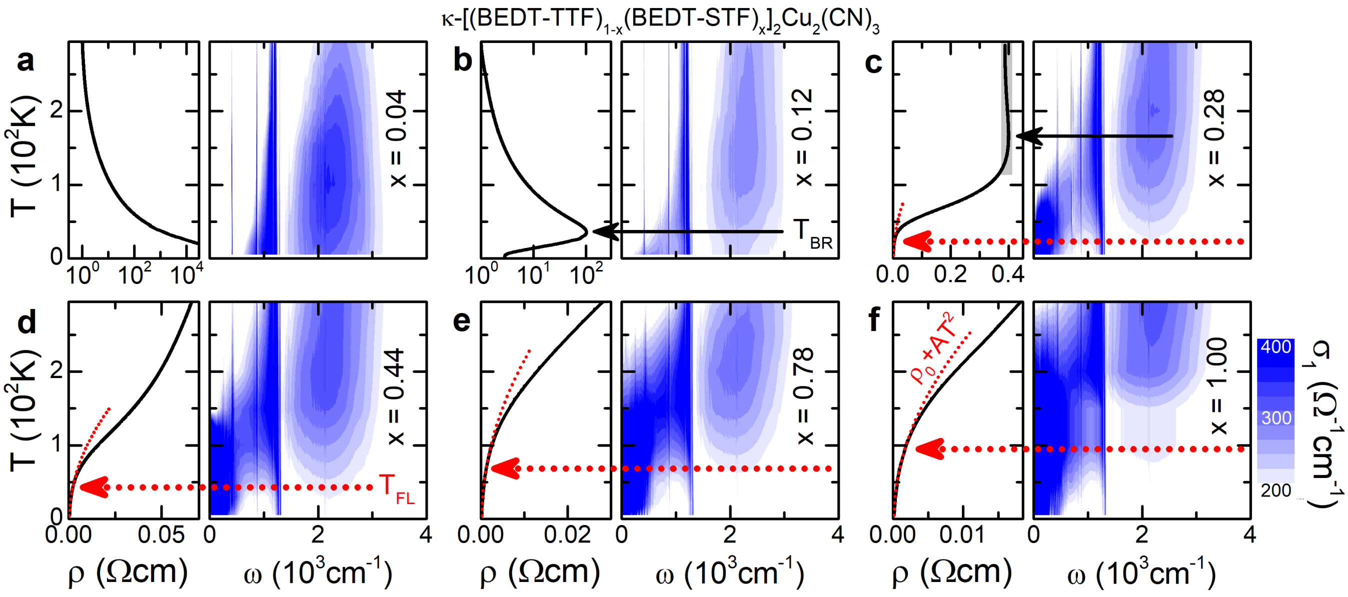

Supplementary Figure 7. **Temperature and frequency evolution of optical conductivity compared to dc resistivity in κ-STF$_x$. a,** The insulating compound with $x = 0.04$ exhibits an increase of the in-gap absorption upon cooling, despite monotonously increasing $\rho(T)$. Such non-thermal behavior of $\sigma_1(T,\omega)$ is also found in the parent compound ($x = 0$) and assigned to metallic fluctuations in the insulating state close to the metal-insulator transition [9]. **b,c,** Below T_{BR} a large part of the spectral weight separates from the Hubbard bands and gradually shifts to lower frequencies upon cooling, where it forms a metallic state at low temperatures. **c-f,** A well-defined quasiparticle peak forms at $T \leq T_{\mathrm{FL}}$. The $\rho \propto T^2$ behavior (red dotted lines) is unanimously linked to a Drude-like response at $\omega = 0$, and the strong increase of resistivity in the bad metal arises from a successive spectral weight shift to finite frequencies upon warming. Metallic behavior is completely lost as the itinerant response merges with the Hubbard bands.

W are determined from the Mott-Hubbard transitions. As indicated in the inset, here we use the half-width at half-maximum on the high-frequency wing for a consistent determination of W because the appearance of the metallic component for the higher substitutions conceals the low-frequency side. The enhancement of W with increasing x substantiates that chemical substitution primarily tunes the bandwidth via the larger transfer integrals of the BEDT-STF molecules. In addition, also U increases, which we assign to the accompanying enlargement of the intra-dimer transfer integrals, that is empirically used to approximate the on-site term via $U \approx 2t_d$ [18, 19]. Overall, the enhancement of W is more pronounced than for U leading to a sizeable reduction of U/W with larger substitution, proving that the main effect of chemical substitution is tuning the bandwidth. This is in excellent agreement with the similarity of the Mott transition observed when applying physical pressure to κ-(BEDT-TTF)$_2$Cu$_2$(CN)$_3$ [6–8]; the results of both experiments are compared in Fig. 4a.

In order to relate the optical response of κ-[(BEDT-STF)$_x$(BEDT-TTF)$_{1-x}$]$_2$Cu$_2$(CN)$_3$ to their transport properties as a function of temperature, in Supplementary Fig. 7 we compare $\rho(T)$ with a false-color contour plot of $\sigma_1(T,\omega)$ for each substitution. For $x \geq 0.28$ one can clearly see that the concentration of optical conductivity around $\omega = 0$ coincides with the T^2-dependence of the resistivity (indicated by dotted red lines) in the Fermi-liquid state. Above T_{FL} the spectral weight associated with metallic quasiparticles gradually shifts to higher frequencies. Since around these temperatures the resistivity also exceeds the Ioffe-Regel-Mott (IRM) limit, the formation of a 'displaced Drude peak' (DDP) is clearly associated with the bad-metal phase. While a DDP was observed in a number of poor electrical conductors subject to pronounced electronic correlations [20], our results conclusively demonstrate that this

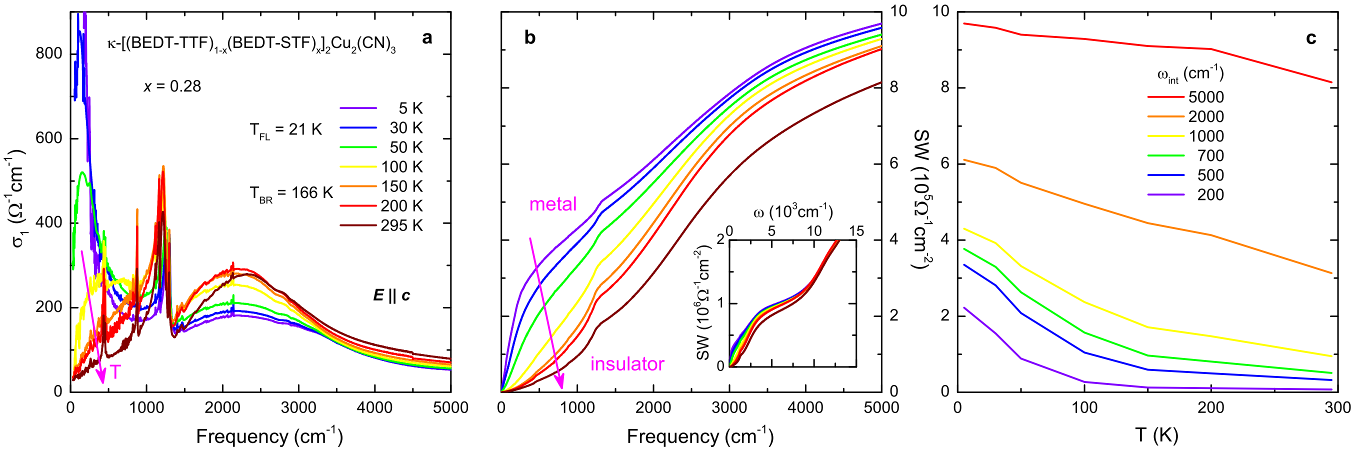

Supplementary Figure 8. **Spectral weight evolution for $x = 0.28$.** **a,** The optical conductivity of κ-[(BEDT-TTF)$_{0.72}$-(BEDT-STF)$_{0.28}$]$_2$Cu$_2$(CN)$_3$ is shown for all temperatures. **b,** The associated spectral weight (SW) clearly indicates the transition from a good metal at low temperatures to an insulating response above $T_{\mathrm{BR}} = 166$ K; in other words spectral weight shifts from low to high frequencies. Inset: for all temperatures the SW is conserved above $\omega \approx 10\,000$ cm^{-1}. **c,** Temperature dependence of the SW integrated up to several distinct cut-off frequencies ω_{int}, as indicated.

phenomenon emerges directly from a Fermi liquid and is, thus, comprised of quasiparticles. This nourishes the idea of 'resilient quasiparticles' discussed recently [21, 22].

The spectral weight shift from low to high frequencies above T_{FL} approximately follows a linear temperature dependence. In Supplementary Fig. 1 we estimated the temperature at which the DDP crosses $\omega = 100$ cm^{-1} (dashed black line) by following the T-ω trajectory of the peak position in the contour plots of Supplementary Fig. 7c-f. As the spectral contribution of itinerant carriers merges with the Hubbard excitations around 2000 cm^{-1}, the metallic properties get lost above T_{BR}, as seen in Supplementary Fig. 7b,c for $x = 0.12$ and 0.28. For the larger substitutions $x \geq 0.44$ this spectral feature broadens increasingly; and considerable weight remains at $\omega = 0$ retaining metallic transport properties. Note, even well above $T = 100$ K, the largest substitutions (Supplementary Fig. 7d-f) exhibit resistivities that deviate by less than a factor two from the low-temperature $\rho \propto T^2$ behavior. Naturally, this broad crossover from a Fermi liquid to a bad metal expresses similarly in $\sigma_1(\omega)$.

Finally, we point out that in Supplementary Fig. 7a-c a 'metallic-like' increase of the low-frequency spectral weight is also observed for $T > T_{\mathrm{BR}}$ where the resistivity exhibits insulating properties, i.e. $\mathrm{d}\rho/\mathrm{d}T < 0$ – even seen for the completely insulating compound with $x = 0.04$. In line with the discussion above, this contribution clearly originates from the metallic properties supporting our previous suggestion [9] that the 'non-thermal' increase of the low-energy spectral weight on the insulating side close to the Mott transition is associated to metallic fluctuations at finite frequencies. Overall, our optical results are in excellent agreement with dc transport, and provide information on the nature of metallic quasiparticles in the bad metal regime far beyond standard resistivity data.

In Supplementary Fig. 8a we exemplify the temperature dependence of the optical conductivity through the various conduction regimes for the example $x = 0.28$. While the 5 K-spectrum comprises a Drude-like peak centered at $\omega = 0$, at temperatures above $T_{\mathrm{FL}} = 21$ K a DDP can be clearly identified; here seen for $T = 30$, 50, and 100 K. As temperature increases, the spectral weight successively shifts to higher frequencies, as seen in the rising intensity around 2000–3000 cm^{-1}. The overall spectral weight, however, remains conserved for all temperatures around $10\,000$ cm^{-1}, above

which other types of excitations become dominant (Supplementary Fig. 8b inset). Panel c illustrates the temperature dependence of the spectral weight, when the integration is conducted from $\omega = 0$ up to different cut-off frequencies ω_{int}; we identify a strong increase below $T_{\rm BR} = 166$ K as metallicity sets in. Clearly, the changes are most pronounced at the lowest frequencies, in particular when $T_{\rm FL}$ is approached.

SUPPLEMENTARY NOTE 4: EXTENDED DRUDE ANALYSIS

The extended Drude model [23, 24] assumes a frequency-dependent scattering rate $\gamma(\omega)$ and effective mass $m^\star(\omega)/m_b$. Both quantities are affected by electronic correlations via $Z < 1$ [25–27]. The complex optical conductivity $\hat{\sigma} = \sigma_1 + i\sigma_2$ of a correlated metal can be written as

$$\hat{\sigma}(\omega) = \frac{\epsilon_0 \omega_p^2}{\gamma} \frac{1}{1 + i\omega(m^\star/m_b)/\gamma}, \tag{1}$$

where $\omega_p = \sqrt{Ne^2/\epsilon_0 m_b}$ is the plasma frequency, reflecting the charge-carrier density N and band mass m_b; ϵ_0 is the permittivity of vacuum and e the elementary charge. Inversion of Supplementary Eq. 1 yields the frequency-dependent scattering rate and effective mass

$$\gamma(\omega) = \epsilon_0 \omega_p^2 \,\mathrm{Re}\left\{[\hat{\sigma}(\omega)]^{-1}\right\} \tag{2}$$

$$\frac{m^\star(\omega)}{m_b} = \frac{\epsilon_0 \omega_p^2}{\omega} \,\mathrm{Im}\left\{-[\hat{\sigma}(\omega)]^{-1}\right\} \quad . \tag{3}$$

The high-energy contributions are summarized as ϵ_∞, which commonly is subtracted from the real part of the complex dielectric constant $\hat{\epsilon} = \epsilon_1 + \mathrm{i}\epsilon_2$ before the extended-Drude analysis can be performed [28]. The particular values of ϵ_∞ range from 2.75 to 3.04 for $x = 0.28$ - 1.00. The plasma frequency ω_p of the conduction electrons in Eqs. (2) and (3) is obtained from the peak of the dielectric loss function $-\mathrm{Im}\left\{\hat{\epsilon}^{-1}\right\} = \epsilon_2/(\epsilon_1^2 + \epsilon_2^2)$, which occurs slightly above 4000 cm^{-1} for the compounds that exhibit metallic transport properties, displayed in Supplementary Fig. 9.

In Supplementary Fig. 10, we plot the effective mass normalized to the band mass, $m^\star/m_b$, determined at the lowest measured temperature ($T = 5$ K) for the four compounds that exhibit Fermi-liquid properties, $x = 0.28$, 0.44, 0.78 and 1.00. Since ω_p shifts only by 5% (Supplementary Fig. 9), m_b is essentially identical for these four substitutions. The frequency dependence of $m^\star(\omega)$ is rather weak in the range 2000–4000 cm^{-1}, i.e. around the Mott-Hubbard transitions. Towards low frequencies, however, $m^\star(\omega)$ exhibits a pronounced increase that eventually saturates at the static limit. This plateau corresponds to the effective mass of long-lived quasiparticles. Overall, our results reveal a frequency dependence of $m^\star/m_b$ similar to the optical data of the paradigmatic Fermi liquid Sr_2RuO_4 (cf. Supplementary Ref. 29, Fig. SM5) and iron pnictides (cf. Supplementary Ref. 30, Fig. 3b); we regard the agreement as a strong confirmation of this concept [25–27, 30–32]. As demonstrated in Fig. 3h, in the low-energy limit ($\omega \to 0$ and $T \to 0$) the effective mass enhancement follows the prefactor to the quadratic frequency dependence of the scattering rate, $\gamma \propto B\omega^2$, while approaching the Mott transition ($x = 1.00 \to 0.28$), yielding $B \propto (m^\star/m_b)^2$ in full accord with the Kadowaki-Woods relation [33].

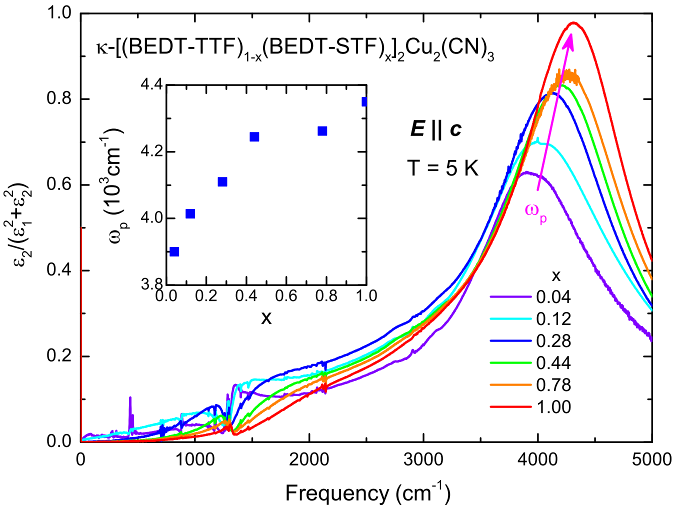

Supplementary Figure 9. **Dielectric loss function of κ-STF$_x$.** The plasma frequency is determined from the peak in the dielectric loss function $-\text{Im}\left\{\hat{\epsilon}^{-1}\right\}$ measured at $T = 5$ K. For all compounds κ-[(BEDT-STF)$_x$(BEDT-TTF)$_{1-x}$]$_2$Cu$_2$(CN)$_3$ we obtain $\omega_p \approx 4000$ cm^{-1}. It slightly increases with substitution indicating a general reduction of the band mass m_b.

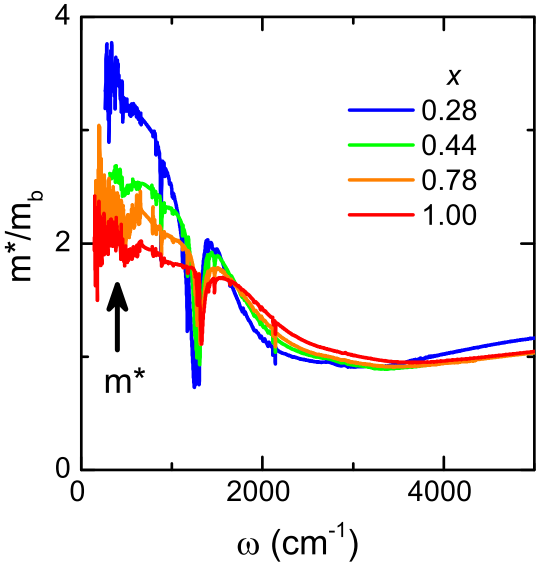

Supplementary Figure 10. **Frequency-dependent effective mass of κ-STF$_x$.** The effective mass $m^\star/m_b$ of κ-[(BEDT-STF)$_x$(BEDT-TTF)$_{1-x}$]$_2$Cu$_2$(CN)$_3$ is determined as a function of frequency by performing an extended Drude analysis. In all cases the low-temperature $m^\star(\omega)/m_b$ increases towards low frequencies where it forms a plateau that corresponds to the quasiparticle mass in the limit $\omega \to 0$.

In Supplementary Fig. 11a-d we display the optical scattering rate $\gamma(T,\omega)$ for the compounds (x = 0.28, 0.44, 0.78, 1.00) that exhibit Fermi-liquid properties in their optical properties. Panels e-h display Gurzhi scaling on the generalized quadratic energy scale $(p\pi k_\text{B}T/\hbar)^2 + \omega^2$ [25–27, 34]. As indicated, we obtained a respective Gurzhi parameter $p = 6 \pm 1$ from our extended-Drude analysis.

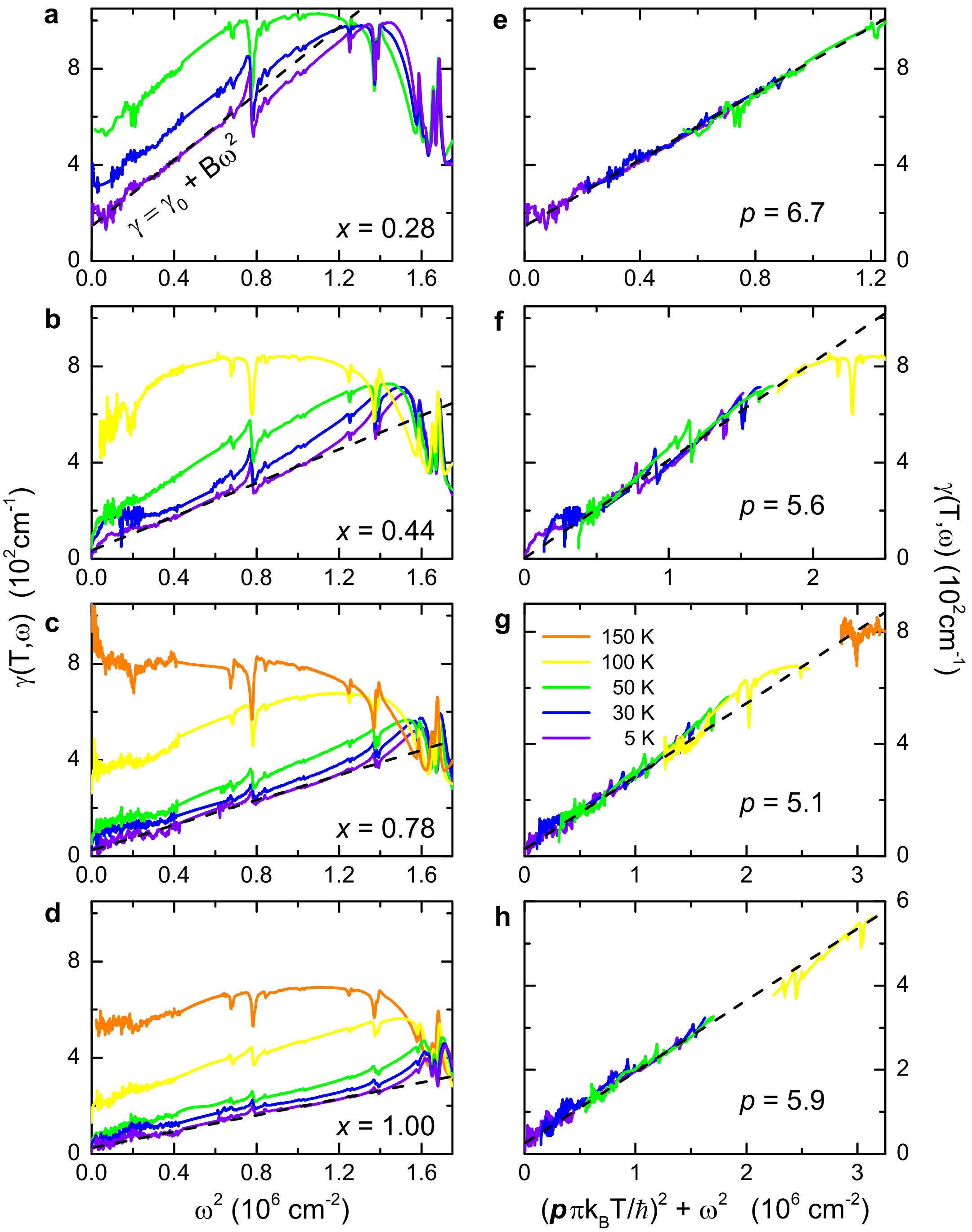

Supplementary Figure 11. ω^2/T^2 **scaling of optical scattering rate. a-d,** $\gamma(T,\omega)$ is displayed for the substitutions $x = 0.28$, 0.44, 0.78, 1.00 on common axes. **e-h,** Renormalized to a generalized energy scale $(p\pi k_B T/\hbar)^2 + \omega^2$. Data are shown in the frequency range where Gurzhi scaling applies.

---

[1] Geiser, U. et al. Superconductivity at 2.8 K and 1.5 kbar in κ-(BEDT-TTF)$_2$Cu$_2$(CN)$_3$: the first organic superconductor containing a polymeric copper cyanide anion. Inorg. Chem. **30**, 2586–2588 (1991). URL `http://dx.doi.org/10.1021/ic00012a005`.

[2] Saito, Y., Minamidate, T., Kawamoto, A., Matsunaga, N. & Nomura, K. Site-specific ^{13}C NMR study on the locally distorted triangular lattice of the organic conductor κ-(BEDT-TTF)$_2$Cu$_2$(CN)$_3$. Phys. Rev. B **98**, 205141 (2018). URL https://link.aps.org/doi/10.1103/PhysRevB.98.205141.

[3] Kandpal, H. C., Opahle, I., Zhang, Y.-Z., Jeschke, H. O. & Valentí, R. Revision of Model Parameters for κ-Type Charge Transfer Salts: An Ab Initio Study. Phys. Rev. Lett. **103**, 067004 (2009). URL https://link.aps.org/doi/10.1103/PhysRevLett.103.067004.

[4] Kanoda, K. & Kato, R. Mott Physics in Organic Conductors with Triangular Lattices. Annu. Rev. Condens. Matter Phys. **2**, 167–188 (2011). URL https://doi.org/10.1146/annurev-conmatphys-062910-140521.

[5] Pustogow, A. et al. Low-Temperature Dielectric Anomaly Arising From Electronic Phase Separation at the Mott Insulator-Metal Transition. npj Quantum Mater. (2021). URL https://doi.org/10.1038/s41535-020-00307-0. arXiv:1907.04437

[6] Kurosaki, Y., Shimizu, Y., Miyagawa, K., Kanoda, K. & Saito, G. Mott Transition from a Spin Liquid to a Fermi Liquid in the Spin-Frustrated Organic Conductor κ-(BEDT-TTF)$_2$Cu$_2$(CN)$_3$. Phys. Rev. Lett. **95**, 177001 (2005). URL https://link.aps.org/doi/10.1103/PhysRevLett.95.177001.

[7] Furukawa, T., Miyagawa, K., Taniguchi, H., Kato, R. & Kanoda, K. Quantum criticality of Mott transition in organic materials. Nat. Phys. **11**, 221–224 (2015). URL http://dx.doi.org/10.1038/nphys3235http://10.0.4.14/nphys3235http://www.nature.com/nphys/journal/v11/n3/abs/nphys3235.html{\#}supplementary-information.

[8] Furukawa, T., Kobashi, K., Kurosaki, Y., Miyagawa, K. & Kanoda, K. Quasi-continuous transition from a Fermi liquid to a spin liquid in κ-(ET)$_2$Cu$_2$(CN)$_3$. Nat. Commun. **9**, 307 (2018). URL https://doi.org/10.1038/s41467-017-02679-7.

[9] Pustogow, A. et al. Quantum spin liquids unveil the genuine Mott state. Nat. Mater. **17**, 773–777 (2018). URL https://doi.org/10.1038/s41563-018-0140-3.

[10] Pinterić, M. et al. Anisotropic charge dynamics in the quantum spin-liquid candidate κ-(BEDT-TTF)$_2$Cu$_2$(CN)$_3$. Phys. Rev. B **90**, 195139 (2014). URL https://link.aps.org/doi/10.1103/PhysRevB.90.195139.

[11] Faltermeier, D. et al. Bandwidth-controlled Mott transition in κ-(BEDT-TTF)$_2$Cu[N(CN)$_2$]Br$_x$Cl$_{1-x}$: Optical studies of localized charge excitations. Phys. Rev. B **76**, 165113 (2007). URL https://link.aps.org/doi/10.1103/PhysRevB.76.165113.

[12] Merino, J., Dumm, M., Drichko, N., Dressel, M. & McKenzie, R. H. Quasiparticles at the Verge of Localization near the Mott Metal-Insulator Transition in a Two-Dimensional Material. Phys. Rev. Lett. **100**, 086404 (2008). URL https://link.aps.org/doi/10.1103/PhysRevLett.100.086404.

[13] Dumm, M. et al. Bandwidth-controlled Mott transition in κ-(BEDT-TTF)$_2$Cu[N(CN)$_2$]Br$_x$Cl$_{1-x}$: Optical studies of correlated carriers. Phys. Rev. B **79**, 195106 (2009). URL https://link.aps.org/doi/10.1103/PhysRevB.79.195106.

[14] Ferber, J., Foyevtsova, K., Jeschke, H. O. & Valentí, R. Unveiling the microscopic nature of correlated organic conductors: The case of κ-(BEDT-TTF)$_2$Cu[N(CN)$_2$]Br$_x$Cl$_{1-x}$. Phys. Rev. B **89**, 205106 (2014). URL https://link.aps.org/doi/10.1103/PhysRevB.89.205106.

[15] Dressel, M. & Drichko, N. Optical properties of two-dimensional organic conductors: signatures of charge ordering and correlation effects. Chem. Rev. **104**, 5689–716 (2004). URL http://apps.webofknowledge.com/full{_}record.do?product=UA{\&}search{_}mode=GeneralSearch{\&}qid=39{\&}SID=T2ogN2hRfrXWYJZq9vq{\&}page=1{\&}doc=1.

[16] Kézsmárki, I. et al. Depressed charge gap in the triangular-lattice Mott insulator κ-(ET)$_2$Cu$_2$(CN)$_3$. Phys. Rev. B **74**, 201101 (2006). URL https://link.aps.org/doi/10.1103/PhysRevB.74.201101.

[17] Elsässer, S., Wu, D., Dressel, M. & Schlueter, J. A. Power-law dependence of the optical conductivity observed in the quantum spin-liquid compound κ-(BEDT-TTF)$_2$Cu$_2$(CN)$_3$. Phys. Rev. B **86**, 155150 (2012). URL https://link.aps.org/doi/10.1103/PhysRevB.86.155150.

[18] Kanoda, K. Recent progress in NMR studies on organic conductors. Hyperfine Int. **104**, 235–249 (1997). URL https://doi.org/10.1023/A:1012696314318.

[19] McKenzie, R. H. A strongly correlated electron model for the layered organic superconductors κ-(BEDT-TTF)$_2X$. Comments Cond. Matt. Phys. **18**, 309 (1998).

[20] Delacrétaz, L., Goutéraux, B., Hartnoll, S. & Karlsson, A. Bad Metals from Fluctuating Density Waves. SciPost Physics **3**, 25 (2017). URL https://scipost.org/SciPostPhys.3.3.025.

[21] Deng, X. et al. How Bad Metals Turn Good: Spectroscopic Signatures of Resilient Quasiparticles. Phys. Rev. Lett. **110**, 086401 (2013). URL https://link.aps.org/doi/10.1103/PhysRevLett.110.086401.

[22] Deng, X., Sternbach, A., Haule, K., Basov, D. N. & Kotliar, G. Shining Light on Transition-Metal Oxides: Unveiling the Hidden Fermi Liquid. Phys. Rev. Lett. **113**, 246404 (2014). URL https://link.aps.org/doi/10.1103/PhysRevLett.113.246404.

[23] Allen, J. W. & Mikkelsen, J. C. Optical properties of CrSb, MnSb, NiSb, and NiAs. Phys.Rev. B **15**, 2952–2960 (1977). URL https://link.aps.org/doi/10.1103/PhysRevB.15.2952.

[24] Dressel, M. & Grüner, G. Electrodynamics of Solids: Optical Properties of Electrons in Matter (Cambridge University Press, Cambridge, 2002). URL http://www.netlibrary.com/urlapi.asp?action=summary{\&}v=1{\&}bookid=73043.

[25] Maslov, D. L. & Chubukov, A. V. First-Matsubara-frequency rule in a Fermi liquid. II. Optical conductivity and comparison to experiment. Phys. Rev. B **86**, 155137 (2012). URL https://link.aps.org/doi/10.1103/PhysRevB.86.155137.

[26] Berthod, C. et al. Non-Drude universal scaling laws for the optical response of local Fermi liquids. Phys. Rev. B **87**, 115109 (2013). URL https://link.aps.org/doi/10.1103/PhysRevB.87.115109.

[27] Maslov, D. L. & Chubukov, A. V. Optical response of correlated electron systems. Rep. Prog. Phys. **80**, 026503 (2017). URL http://stacks.iop.org/0034-4885/80/i=2/a=026503?key=crossref.e162a422a646a2aec909b8493a4387ba.

[28] van Heumen, E. et al. Optical and thermodynamic properties of the high-temperature superconductor $HgBa_2CuO_{4+\delta}$. Phys.Rev. B **75**, 054522 (2007). URL https://link.aps.org/doi/10.1103/PhysRevB.75.054522.

[29] Stricker, D. et al. Optical Response of Sr_2RuO_4 Reveals Universal Fermi-Liquid Scaling and Quasiparticles Beyond Landau Theory. Phys. Rev. Lett. **113**, 087404 (2014). URL https://link.aps.org/doi/10.1103/PhysRevLett.113.087404.

[30] Tytarenko, A., Huang, Y., de Visser, A., Johnston, S. & van Heumen, E. Direct observation of a Fermi liquid-like normal state in an iron-pnictide superconductor. Sci. Rep. **5**, 012421 (2015). URL https://doi.org/10.1038/srep12421http://10.0.4.14/srep12421https://www.nature.com/articles/srep12421{\#}supplementary-information.

[31] Nagel, U. et al. Optical spectroscopy shows that the normal state of URu_2Si_2 is an anomalous Fermi liquid. Proc. Natl. Acad. Sci. **109**, 19161–19165 (2012). URL http://www.pnas.org/content/109/47/19161.abstract.

[32] Mirzaei, S. I. et al. Spectroscopic evidence for Fermi liquid-like energy and temperature dependence of the relaxation rate in the pseudogap phase of the cuprates. Proc. Natl. Acad. Sci. **110**, 5774 (2013). URL http://www.pnas.org/content/110/15/5774.abstract.

[33] Jacko, A. C., Fjaerestad, J. O. & Powell, B. J. A unified explanation of the Kadowaki-Woods ratio in strongly correlated metals. Nat. Phys. **5**, 422–425 (2009). URL http://dx.doi.org/10.1038/nphys1249http://www.nature.com/nphys/journal/v5/n6/suppinfo/nphys1249{_}S1.html.

[34] Gurzhi, R. Mutual Electron Correlations in Metal Optics. J. Exp. Theor. Phys. **8**, 673 (1959). URL http://www.jetp.ac.ru/cgi-bin/e/index/e/8/4/p673?a=list.
